# Raising awareness on the therapeutic role of cholecalciferol in CKD: a multidisciplinary-based opinion

**DOI:** 10.1007/s12020-017-1369-3

**Published:** 2017-07-19

**Authors:** Sandro Giannini, Sandro Mazzaferro, Salvatore Minisola, Luca De Nicola, Maurizio Rossini, Mario Cozzolino

**Affiliations:** 1 Department of Medicine, Clinica Medica 1, University of Padova, Padova, Italy; 2 Department of Cardiovascular Respiratory Nephrologic Anesthetic and Geriatric Sciences, Sapienza University of Rome, Rome, Italy; 3 Department of Internal Medicine and Medical Disciplines, Sapienza University of Rome, Rome, Italy; 4 Division of Nephrology, Second University of Naples, Naples, Italy; 5 Department of Medicine, Rheumatology Unit, University of Verona, Verona, Italy; 6 Department of Health Sciences, Renal Division and Laboratory of Experimental Nephrology, San Paolo Hospital, University of Milan, Milan, Italy

**Keywords:** Vitamin D, Cholecalciferol, Chronic kidney disease, Proteinuria, Consensus statement, Cardiovascular disease, Bone disease

## Abstract

Vitamin D is recognized to play an essential role in health and disease. In kidney disease, vitamin D analogs have gained recognition for their involvement and potential therapeutic importance. Nephrologists are aware of the use of oral native vitamin D supplementation, however, uncertainty still exists with regard to the use of this treatment option in chronic kidney disease as well as clinical settings related to chronic kidney disease, where vitamin D supplementation may be an appropriate therapeutic choice. Two consecutive meetings were held in Florence in July and November 2016 comprising six experts in kidney disease (*N* = 3) and bone mineral metabolism (*N* = 3) to discuss a range of unresolved issues related to the use of cholecalciferol in chronic kidney disease. The panel focused on the following six key areas where issues relating to the use of oral vitamin D remain controversial: (1) vitamin D and parathyroid hormone levels in the general population, (2) cholecalciferol in chronic kidney disease, (3) vitamin D in cardiovascular disease, (4) vitamin D and renal bone disease, (5) vitamin D in rheumatological diseases affecting the kidney, (6) vitamin D and kidney transplantation.

## Introduction

Hypovitaminosis D is a major health problem, with a prevalence ranging from 33 to 58% in the general population [1–3]. Several recognized negative health outcomes are associated with low vitamin D status, such as cardiovascular disease (CVD), diabetes, malignant and autoimmune diseases, bone fractures and increased mortality [4, 5]. In patients with chronic kidney disease (CKD), alteration in vitamin D metabolism plays a central role in the development of secondary hyperparathyroidism (SHPT), in addition to being associated with increased CV morbidity and mortality in these patients [6]. A hallmark of vitamin D insufficiency/deficiency is elevated levels of parathyroid hormone (PTH). Vitamin D plays an important role in maintaining calcium and phosphate homeostasis and as a consequence the majority of guidelines on the use of vitamin D in CKD have largely been based on levels of PTH and calcium [1, 7]. Most of circulating vitamin D (vitamin D_3_, cholecalciferol) derives from the photolysis of 7-dehydroxycholesterol, which occurs in the skin upon sunlight exposure [8]. Indeed, UVB exposure has been proved effective in increasing the serum levels of 25-hydroxyvitamin D (25-OH-D) in patients with end-stage kidney disease on dialysis [9] and in CKD patients on dialysis and healthy subjects receiving 20 µg/day cholecalciferol [10]. However, as sunlight exposure depends on the latitude and season and only a small fraction of circulating vitamin D_3_ originates from dietary intake, vitamin D supplementation is often required. Since the late 70’s, native vitamin D and nonselective vitamin D receptor (VDR) activators have been used mainly for lowering of PTH levels. In the past two decades selective VDR activators have gained recognition for their importance in the management of CKD-mineral bone disease (MBD) [11, 12] and as such are considered standard therapy in these patients [12].

More recently, vitamin D deficiency has been linked to a whole host of diseases, some related to CKD, prompting further exploration of the mechanism of action of vitamin D analogs and, consequently, their potential benefit in clinical trials [13].

However, many open questions regarding the use of native vitamin D or VDR activators remain [14–17].

First, the K/DOQI and KDIGO guidelines recommend testing for vitamin D insufficiency and deficiency in CKD patients, however, no consensus on the definition of vitamin D insufficiency in CKD is currently available [18–20].

Second, the question regarding the use of native vitamin D in patients with CKD remains heavily debated, particularly with regard to the choice of compound, when to treat, and the most suitable dose to administer with or without VDR activators [15, 21–23] (Table 1). The aim of this review was to discuss a range of unresolved issues related to the use of the native vitamin D cholecalciferol in CKD. The following six key areas relating to the use of native vitamin D and that remain controversial were highlighted: (1) vitamin D and PTH levels in the general population, (2) cholecalciferol in CKD, (3) vitamin D in CVD, (4) vitamin D and renal bone disease, (5) vitamin D in rheumatological diseases affecting the kidney, (6) vitamin D and kidney transplantation.

**Table 1:** Vitamin D and VDR activators currently available

Name	Source/synthesis	Active	Selective	Molecule	Molecular formula, ATC code
Cholecalciferol	Sunlight exposure and diet	No	No	Vitamin D_3_	C_27_H_44_O, A11CC05
Ergocalciferol	Diet	No	No	Vitamin D_2_	C_28_H_44_O, A11CC01
Calcidiol	Mainly produced from first hydroxylation of vitamin D in liver	No	No	25-hydroxyvitamin D_3_	C_27_H_44_O_2_, A11CC06
Calcitriol	Mainly produced from second hydroxylation of vitamin D in kidney. Binds VDR directly	Yes	No	1,25-dihydroxyvitamin D_3_	C_27_H_44_O_3_, A11CC04
Alfacalcidol	Synthetic analog of calcitriol, converted to calcitriol in liver before binding to VDR	No	No	1 α-hydroxyvitamin D_3_	C_27_H_44_O_2_, A11CC03
Paricalcitol	Acts as synthetic agonist to VDR. Not converted to calcitriol before binding	Yes	Yes	19-NOR-1 α dihydroxyvitamin D_2_	C_27_H_44_O_3_, H05BX02
Maxacalcitol	Acts as synthetic agonist to VDR. Not converted to calcitriol before binding	Yes	Yes	22-oxa-1,25-dihydroxyvitamin D_3_	C_27_H_44_O_4_, N/A

*VDR*  vitamin D receptor

## Vitamin D and PTH levels in the general population

The relationship between vitamin D and PTH in normal subjects has been the issue of intensive investigations over the past decade. This relationship is important because if we use serum 25-OH-D values at which the serum PTH levels are suppressed as a marker of vitamin D sufficiency, therapeutic implications are clear.

At least two 25-OH-D levels have been proposed [24]. The 50 nmol/L (20 ng/mL) threshold is mainly supported by the Institute of Medicine (it is probably more suitable for the general population). The 75 nmol/L (30 ng/mL) is supported by the Endocrine Society and other European Societies (it is probably targeted to patients).

It is important to note that there are several problems in calculating these cut-off values; most notably, these include statistical methodology, inability to account for confounding factors, and methods of measurement of the two variables (i.e., 25-OH-D and PTH). As a result, thresholds ranging from 12 to 50 ng/mL or no threshold are reported in the literature [25, 26].

A number of factors or clinical conditions can influence serum levels of PTH in subjects with normal renal function. Some can be easily recognized, such as for example the use of drugs (i.e., thiazide diuretics, and lithium); other factors or conditions might be difficult to identify, unless specifically investigated [27]. The most notable example is represented by vitamin D insufficiency. Indeed, since serum PTH levels decrease following vitamin D administration [28], it derives that patients who are insufficient are in general characterized by higher serum hormone levels.

It is well known that with declining renal function, there is a rise in serum PTH. Most authorities believe that this increase begins when estimated glomerular filtration rate (eGFR), calculated by MDRD or the CKD EPI formula, is <60 mL/mm/1.73 m^2^ [29]. However, these values may be found in apparently healthy normal subjects, particularly in the ageing population. In a recent large cross-sectional study on 1128 subjects with normal renal function stratified by eGFR levels, PTH significantly increased with decreasing eGFR levels [30], in line with a previous prospective study conducted in 1664 stage three CKD patients [31]. Interestingly, in subjects with normal renal function, the rise of plasma PTH has been shown to precede the increase of plasma fibroblast-growth factor 23 (FGF23), an independent marker of poor renal and CV outcomes and mortality in CKD, whereas, in the course of CKD, whether PTH or FGF23 rises earlier depends on the status of vitamin D [31].

Theoretically, there are a number of other factors that, in addition to vitamin D status and kidney function, may influence PTH concentrations in normal individuals. For example, race (black vs. white), weight (lean vs. obese) [25], calcium intake and age are the most well documented in the literature. These differences may partly or fully disappear when a state of vitamin D sufficiency is reached, suggesting that these factors act through the vitamin. However age, per se, seems to be independently associated with higher PTH values [27, 32].

## Questions and answers

### 1. Is there any level of 25-OH-D above which there is no longer PTH suppression**?**

There is no straightforward answer to this question. As outlined previously, there are a number of issues that could affect the calculation of this cut-off value. For example, a recent study [27] investigated the relationship between calcidiol and PTH concentration using the LOWESS representation. No clear inflection point was detected, i.e., a 25-OH-D concentration above which PTH no longer decreases. This dispute, dating back to the beginning of this century [33, 34] also applies to patients with renal failure, where studies addressing this issue are scanty.

### 2. Should we measure vitamin D levels in presumptive normal subjects to eliminate one reason for spurious increases in PTH levels?

The most recent guidelines on the diagnosis and management of asymptomatic primary hyperparathyroidism recommend excluding subjects with low 25-OH-D values from a reference population, when determining serum PTH normal values [35, 36]. This is mainly attributed to the fact that several studies are available showing that increasing calcidiol values to 30 ng/mL lowers serum PTH by about 20–35%, depending on the method used to assay the hormone [27, 37]. We therefore suggest measuring calcidiol values (using the 30 ng/mL threshold) because this increases the specificity of serum PTH measurement, thus eliminating one of the possible causes of hormone elevation.

### 3. Should subjects be excluded with eGFR values <60 mL/min/1.73 m^2^ to obtain normal values? In addition, is the calculation of GFR a prerequisite parameter to obtain normative data of serum PTH values? Should we take other parameters into account?

Studies in subjects with a eGFR rate >60 mL/min/1.73 m^2^ are recommended if we want to derive reference values for PTH [38]. A recent study showed that when enrolling subjects with these biochemical features together with 25-OH-D values > 30 ng/mL, the upper value of PTH was 22.4 lower than the upper limit suggested by the manufacturer [39]. We therefore suggest including only subjects with normal kidney function (i.e., eGFR >60 mL/min/1.73 m^2^) when looking at PTH reference values.

Another important issue to be considered is represented by factors known to influence PTH secretion. It is indeed well-known that, for example, serum PTH values are higher in black than in white population [40], in obese subjects vs. lean individuals [41] and in elderly compared to young [42]. We suggest taking into account vitamin D status and kidney function when deriving normal PTH values. There are less consistent data in other areas; furthermore, some factors (i.e., obesity and the black ethnicity) may act through calcidiol status. As soon as compelling data are available a more accurate definition of PTH normal values can be achieved. More importantly, the comparison with whatever reference range is used (taking into account both Vitamin D status and kidney function) must be easily achievable in clinical practice.

### Cholecalciferol therapy in CKD

The pleiotropic effects of vitamin D fit well with the potential benefits of multifactorial nephrology care in CKD in non-dialysis as in dialysis setting [43–46]. To date, however, we have no solid data, that is, generated by well-designed controlled trials, demonstrating that the administration of native vitamin D in CKD patients ameliorates “hard” clinical outcomes besides and beyond the metabolic profile [47–51]. However, in the next future, some trials in CKD currently listed on “ClinicalTrials.gov” will probably provide information on the effects of vitamin D on cardiac hypertrophy (NCT01323712), insulin resistance (NCT00893451), eritropoyetin dosage (NCT01395823), proteinuria (NCT01426724), and maturation of the arteriovenous fistula for dialysis (NCT00912782).

While awaiting for these studies, clinical nephrologists eagerly look forward to suggestions aimed at improving their practice. In this respect, “reasonable reasoning” on (still) unmet questions on the extraskeletal indications of cholecalciferol is helpful.

## Questions and answers

### 1. Can we consider cholecalciferol as part of an antiproteinuric intervention in CKD?

Residual proteinuria after therapy with maximal tolerated doses of renin-angiotensin system (RAS) blockers is widely recognized as the major independent risk factor for progressive GFR loss and CV events in non-dialysis CKD patients [52–55]. Recent evidence supports the use of vitamin D acts as “the good companion” of anti-RAS agents, or as an alternative option in patients intolerant to these drugs. Indeed, clinical studies and supporting mechanistic studies suggest that vitamin D deficiency contributes to inappropriate activation and/or lack of adequate suppression of RAS in renal disease, while exogenous administration decreases transcription of renin as well as of several pro-inflammatory factors either linked or not to RAS [56, 57]. Early clinical studies have highlighted the efficacy of active vitamin-D forms in reducing proteinuria in CKD patients under anti-RAS therapy, with calcitriol reducing proteinuria in the early stages of CKD, while paricalcitol has also been shown to be efficacious in more advanced stages [58–62]; however, these studies have highlighted hypercalcemia as a common side effect of either agent. More recently, three studies have renewed interest on cholecalciferol as a safe antiproteinuric agent in CKD [63–65].

A study from the Imperial College in London evaluated the effects of high-dose cholecalciferol supplementation in 52 type 2 diabetic patients with albuminuria (urine albumin creatinine ratio-ACR >30 mg/mmol), despite established anti-RAS therapy, and vitamin D deficiency (serum 25-OH-D ≤16 ng/mL) or insufficiency (25-OH-D 16–32 ng/mL) [63]. Patients with vitamin D deficiency were treated with oral cholecalciferol 40,000 units weekly in the first 2 months and monthly thereafter, while those with insufficiency were treated with same dose but on a monthly basis since the beginning of therapy. After 4 months of therapy, in the absence of changes of serum calcium, 78% patients achieved the replete status of 25-OH-D (≥30 ng/mL) and, in parallel, ACR decreased by 25.6%. Interestingly, a concomitant significant reduction in TGF-ß1 levels was also observed, suggesting a nephroprotective effect of cholecalciferol mediated at least in part by the suppressed release of this well recognized profibrotic cytokine [66]. These data were only in part confirmed by a later study in Chinese type 2 diabetic patients with nephropathy [64]. Authors showed that a rise in vitamin D concentrations after therapy with lower doses of cholecalciferol (800 IU/day) associated with only a slight and transitory improvement in proteinuria; however, the study was limited by the small size of treated patients (*n* = 22).

Recently, a prospective controlled study has generated more solid data on the antiproteinuric efficacy of conventional doses of cholecalciferol [65]. Molina et al. enrolled 101 Caucasian non-dialysis CKD patients (mean age of 73 years, eGFR 39 mL/min/1.73 m^2^, ACR 254 mg/g). Those with low 25-OH-D (17 ng/mL on average) and SHPT (iPTH 125 pg/mL on average) received oral cholecalciferol (666 IU/day), whereas those without hyperparathyroidism, independent of their vitamin D status, did not receive any cholecalciferol, and were used as controls. The main finding that emerged from this study was that by month 6 cholecalciferol had increased 25-OH-D levels by 53% and concurrently decreased albuminuria by 41%, while no improvement was observed in the control group [65].

Therefore, experimental evidence and observational clinical studies support the use in clinical practice of cholecalciferol as adjuvant of (or alternative to) anti-RAS agents to lower proteinuria.

### 2. Can cholecalciferol contribute to ameliorating renal anemia?

The new goal of antianemic therapy in CKD is abating erythropoietin stimulating agent (ESA) hyporesponsiveness [67, 68]. Indeed, this condition, defined as a failure to achieve target hemoglobin (Hb) levels despite a higher-than-usual dose of ESA or a continuous need for a higher dose to maintain these levels [67], is a major complication of CKD because: (I) it is present in up to 25% of patients, (II) affects non-dialysis as dialysis patients, (III) heralds a poor cardiorenal prognosis [67–70]. Modifiable determinants of ESA hyporesponsiveness are several, the main being iron deficiency; however awareness is now growing on the role of vitamin D. Indeed, across the whole spectrum of CKD, vitamin D deficiency consistently worsens anemia and increases resistance to ESA, while exogenous administration of native and active forms allows improvement of anemia with a reduction of ESA dosing likely due to the antinflammatory and PTH-suppressing properties [62, 71–75].

In particular, a 1-year prospective study in 158 dialyzed patients, on the effects of cholecalciferol supplementation on mineral metabolism, inflammation and cardiac parameters, and correction of vitamin D deficiency allowed a parallel significant decline of darbepoietin dose and C-reactive protein level, suggesting amelioration of inflammatory status as the beneficial effect underlying improved response to ESA after cholecalciferol therapy [51]. Interestingly, in the population under study, 25-OH-D level was less than 30 ng/mL in almost 80% patients at baseline, and, after 6 months of supplementation, the level showed a marked increase, from 22.3 to 42.0 ng/mL on average. This finding supports the appropriateness of cholecalciferol dosing chosen by Authors that was based on the severity of vitamin D insufficiency, that is, 50,000 IU once a week if 25-OH-D levels were <15 ng/mL, 10,000 IU once a week when level was between 16 and 30 ng/mL, and 2700 IU three times per week when levels were >30 ng/mL.

In contrast to supplementation with cholecalciferol, ergocalciferol replenishment has been shown to exert no effect on the use of epoetin in 276 dialyzed patients with serum 25-OH-D level <30 ng/mL, in a double-blind, placebo-controlled randomized trial [76]. Indeed, ergocalciferol supplementation for 6 months did not produce any significant change in epoetin dose in either ergocalciferol or placebo arm, or between them, despite increased 25-OH-D levels [76].

### 3. What is the best way to administer cholecalciferol in CKD patients?

Current clinical practice guidelines do not provide indications on the use of native vitamin D in dialysis; however, they do recommend measuring 25-OH-D in non-dialysis patients with rising PTH level, and, in the case of 25-OH-D level <30 ng/mL, they suggest to consider first native vitamin D while leaving the active, and more expensive, analogs as a second option [19, 77]. This concept has been reinforced by the publication of results of two recent trials in stage 3–5 non-dialyisis CKD patients, that have failed to demonstrate improved CV protection of paricalcitol vs. placebo [78, 79]. On the other hand, these trials have disclosed a higher risk of hypercalcemia in the paricalcitol arm. This safety issue has been raised also in recent meta-analyses supporting the hypercalcemia risk associated with calcitriol and vitamin D analogs [80, 81]. Most likely, the updated KDIGO guidelines on CKD-MBD, expected to be published by the end of 2017, will take into account these new data. While “sufficient” consensus has been reached on treating CKD patients with low vitamin D status (i.e., 25-OH-D <30 ng/mL), and start vitamin D therapy by prescribing native forms, the optimal modality (dosing and schedule) still remains unknown. Nevertheless, some hints can be provided.

In maintenance hemodialysis patients with 25-OH-D levels <30 ng/mL, oral weekly administration of 25,000 IU of cholecalciferol has been recently proven to be an effective, safe, and inexpensive way to replete vitamin D status [82]. However, the study also showed that this therapy does not lower PTH levels, suggesting that higher doses are needed in the dialysis population [83]. The picture becomes more complex in non-dialysis patients where endogenous production of vitamin D, and resistance to its effects as well, are more heterogeneous, due to the intrinsic variability of renal damage in this setting.

A recent systematic review addressed the effects of vitamin D supplementation, in the form of ergocalciferol or cholecalciferol, on various health outcomes in early CKD [84]. Two results of the study merit attention: (I) cholecalciferol is more effective than ergocalciferol in raising and maintaining 25-OH-D concentrations, (II) a minimum daily dose of 2000 IU of cholecalciferol (equivalent to 14,000 IU/week) is required to correct vitamin D deficit.

Therefore, it is reasonable to suggest a weekly supplementation (better than daily administration to allow proper adherence to prescription) of cholecalciferol at starting doses in the 14,000–25,000 range (higher in dialysis than in non-dialysis patients). Subsequent titration of drug dosing should be individualized and not based only on 25-OH-D levels, but also on main surrogates of outcome (PTH, proteinuria, ESA responsiveness). Of note, supplements should be withheld if serum 25-OH-D level is >100 ng/mL or serum calcium level is >10.5 mg/dL.

As an additional piece of the already intricate puzzle of vitamin D therapy, it is worth mentioning that some Authors are proposing a combined intervention, that is, with cholecalciferol plus active analogs, to optimize control of the abnormalities of vitamin D metabolism in CKD [16, 85]. A recent observational study in hemodialyzed patients supports this novel approach by showing that a combination of low doses of cholecalciferol (5000 IU/week) and paricalcitol (10 μg/week) allows significant amelioration of biochemical MBD parameters (25-OH-D and PTH levels), with no side effects [86]. Effectiveness of dual therapy should be tested in the future by formal trials given the solid rationale and the potential reduction of costs and side effects related to high-dose therapy with active analogs of vitamin D.

**Fig. 1:**
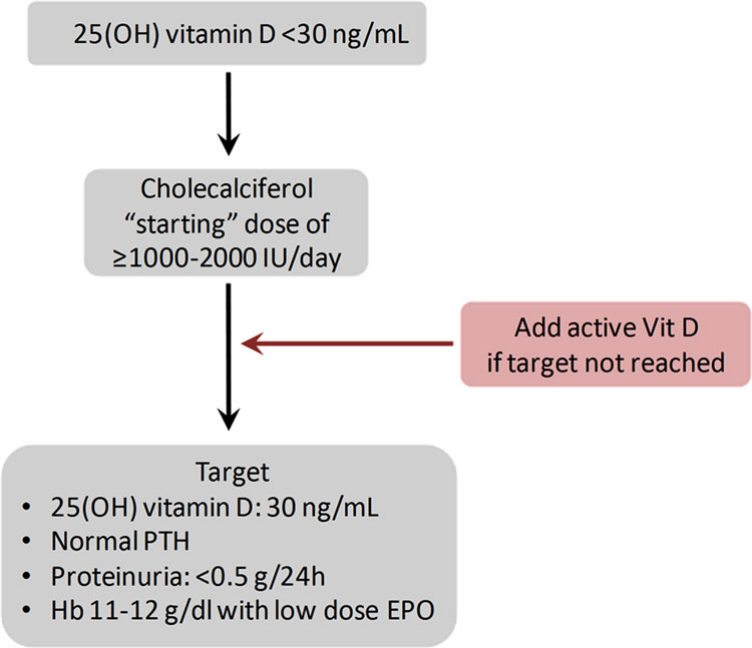
Suggested algorithm for cholecalciferol therapy in CKD patients with low 25-OH-D levels. Dose of cholecalciferol is considered a starting dose. Abbreviations: *Vit D* vitamin D, *PTH* parathyroid hormone, *Hb* hemoglobin, *EPO* erythropoietin

Therefore, in early as in advanced CKD, the clinical goal of vitamin D therapy is to increase 25-OH-D levels to normal in order to optimize control of proteinuria, anemia and PTH. In the presence of low endogenous 25-OH-D levels, it is reasonable to start supplementation with cholecalciferol, while active analogs can be added if therapeutic goals are not met (Fig. 1).

### Vitamin D in CVD

Several studies have suggested a possible role of vitamin D deficiency as a risk factor in the pathogenesis of CVD in the general population as well as in CKD patients. Inappropriate activation of the RAS plays a central role in the regulation of blood pressure, electrolyte, and volume homeostasis and may represent a major risk factor for hypertension, heart attack and stroke [87]. Furthermore, vitamin D deficiency can contribute to an inappropriately activated or unsuppressed RAS [88]. VDR knockout mice show a marked increase in myocardial renin expression, plasma Angiotensin II production, hypertension and cardiomyocyte hypertrophy [88, 89]. Vitamin D regulation of renin expression was found to be independent of calcium metabolism and 1,25(OH)2 D3 markedly suppressed renin transcription by a VDR-mediated mechanism in cell cultures. Hence, 1,25(OH)2 D3 is recognized as a novel negative endocrine regulator of the RAS [88]. Evidence from clinical studies has demonstrated an inverse relationship between circulating vitamin D levels and the blood pressure and/or plasma renin activity, but the mechanism is not yet fully understood [90].

It is therefore suggested to test for and treat low 25-OH-D concentrations with vitamin D supplementation to suppress the detrimental effects of angiotensin II on CV system.

## Questions and answers

### 1. Is vitamin D deficiency related to cardiovascular events and mortality?

Despite the loss in renal mass with progressive CKD, there has been renewed interest in studying the mineral effects of supplementation with calciferols in CKD patients with low vitamin D levels. This interest has been amplified by studies that have demonstrated several potential extra-skeletal benefits of vitamin D [91]. Vitamin D deficiency is associated with albuminuria and a higher prevalence of CV disease and mortality in the Third National Health and Nutrition Examination Survey (NHANES III) cohort [92, 93]. Several other studies have shown an association between vitamin D deficiency and other traditional CV risk factors such as hypertension, insulin resistance, diabetes, and dyslipidemia [94, 95]. A recent umbrella review of systematic reviews and meta-analyses of observational studies and randomized trials demonstrated suggestive evidence for a correlation between high vitamin D concentrations and low risk of CVD, CVD prevalence, hypertension, ischemic stroke and stroke [96]. Yet, an association between mortality and vitamin D deficiency has been shown in dialysis- and nondialysis-dependent CKD [92, 97]. Because the risk of CVD is higher in CKD than non-CKD patients, the potential benefits of vitamin D supplementation (D3-cholecalciferol supplementation) may be greater in CKD than in non-CKD individuals [98]. Associations between low 25-OH-D level and albuminuria, a known CV risk factor, have been shown in recent studies [93, 99, 100]. Intervention studies using active vitamin D analogs have shown a reduction in proteinuria among CKD patients [101].

In conclusion, to date, no clear-cut evidence exists supporting the hypothesis that native vitamin D treatment and the correction of 25-OH-D hypovitaminosis are able to lower the risk of mortality and CV events. Therefore, no recommendations but only suggestions can be made that vitamin D supplementation may improve CV mortality and decrease the risk of CV events.

### 2. Do clinical findings support preclinical data that the correction of vitamin D deficiency has an impact on blood pressure control and/or plasma renin activity?

Vitamin D deficiency affects CV disease through the action of several factors including endothelial dysfunction and RAS activation. Endothelial dysfunction is an early subclinical sign of CV disease, and it participates in the underlying etiology of premature atherosclerosis. Hormonal abnormality, including RAS activation, is a contributor to the development of essential hypertension. In experimental models, vitamin D insufficiency or deficiency contributed to high blood pressure through RAS activation, which resulted in ventricular hypertrophy and therefore the deterioration of cardiac function [89]. By regulating RAS, calcitriol administration not only normalized blood pressure but also restored cardiac function. Scragg et al. identified, in the third NHANES, that an inverse relationship existed between 25-OH-D and systolic blood pressure, and this relationship remained significant even after adjusting for age, sex, ethnicity, physical activity, and body mass index. Individuals in the highest quintile had lower systolic blood pressure than those in the lowest 25-OH-D quintile [102].

Furthermore, the effect of 8 weeks of supplementation with vitamin D plus calcium, compared to calcium-only supplementation, on the blood pressure among 148 women aged 70 years or older was studied. Vitamin D plus calcium significantly lowered systolic blood pressure by 9.3%, and the calcium-only supplementation lowered it by 4.1% compared to baseline [103].

Several publications have favored the beneficial CV effects after supplementation with vitamin D. A trial in the United States randomly assigned 283 African American subjects into a 4-arm, double-blind trial of placebo, 1000, 2000, or 4000 IU of oral cholecalciferol per day. At baseline and at 3 months, the systolic and diastolic blood pressure and 25-OH-D levels were measured. This study found that although cholecalciferol supplementation did not affect diastolic pressure (*P* = 0.37), the difference in systolic pressure between baseline and 3 months was +1.7 mmHg for those receiving placebo, −0.66 mmHg for 1000 IU/day, −3.4 mmHg for 2000 IU/day, and −4.0 mmHg for 4000 IU/day of cholecalciferol (−1.4 mmHg for each additional 1000 IU/day of cholecalciferol; *P* = 0.04). For each 1 ng/mL increase in plasma 25-OH-D, there was a significant 0.2 mmHg reduction in systolic pressure (*P* = 0.02) [104].

Larsen et al. investigated the effect of 3000 IU vitamin D per day for 20 weeks in a randomized, placebo-controlled, double-blind study in 130 hypertensive patients residing in Denmark [105]. Vitamin D supplementation reduced the systolic pressure significantly. In a post-hoc subgroup analysis of 92 subjects with baseline 25-OH-D levels <32 ng/mL, significant decreases in the 24 h systolic and diastolic BP were observed in response to cholecalciferol supplementation [105]. Moreover, suggestive evidence on the reduced risk of hypertension upon vitamin D supplementation was also recently provided in an umbrella review of systematic reviews and meta-analyses of observational and randomized studies [96]. Finally, suberythematous doses of UVB but not UVA, thrice weekly for 6 weeks, were shown to significantly reduce the 24-h systolic and diastolic blood pressure in patients with untreated mild hypertension [106]. Notably, UVB but not UVA exposure also increased plasma 25-OH-D levels by 162% and decreased iPTH by 15% [106].

Yet, despite the large number of clinical studies that have been conducted to examine the effect of vitamin D supplementation on blood pressure, there is still no clear consensus on the potential antihypertensive effect of vitamin D.

### 3. Can vitamin D correction reduce CV morbidity and mortality?

A recent meta-analysis including 75,927 participants from 38 trials (mostly healthy) demonstrated that cholecalciferol supplementation could decrease mortality in elderly people living independently or in institutional care [107]. However, the results obtained in the umbrella review from Theodoratou and coworkers failed to draw conclusions on the relation between vitamin D and CVD mortality or mortality in CKD [96].

Therefore, further trials are warranted on this specific important issue.

## Vitamin D and renal bone disease

Renal patients suffer an increased rate of bone fractures, responsible for morbidity and mortality. Histological lesions in renal patients, identified as renal osteodystrophy (ROD), include any of the following: osteitis fibrosa, osteomalacia, mixed and adynamic bone (ABD). Besides mechanical competence, also the metabolic role of bone is disturbed with hampered buffering capacity of divalent ions and associated increased risk of ectopic (vascular) calcifications. This increased risk seems especially true for ABD, which is specifically regarded as a clinical condition of increased morbidity and mortality from fractures and CVD [108].

More recently, this histologic classification of ROD, although regarded as standard, has been challenged and a new one has been suggested focused on the three parameters most probably affecting clinical outcomes, i.e., bone Turnover, Mineralization and Volume (so called TMV classification) [48]. Theoretically, these parameters should represent specific therapeutic targets, but nephrologists acknowledge a limited knowledge of ROD [109] since bone biopsies are rarely performed in renal patients and are mostly limited to the very late stages of the disease. In recent years, the increased appreciation of renal insufficiency in the general aging population raised the question of the occurrence of osteoporosis (OP) in aged renal patients [110]. OP is a condition of reduced bone strength favoring fractures, a definition applicable also to ROD. However, OP results from the prevailing of bone resorption on formation producing a progressive loss of bone volume and density but with normal mineralization. At variance, lesions of ROD are quite different and also include abnormal bone texture and mineralization. Therefore, while OP can be easily diagnosed by means of the measurement of bone mineral density (BMD; mostly through DXA scan), this is not true for ROD [77] since low values can be encountered even in the absence of OP. However, recent clinical observations have specifically demonstrated that a reduced BMD in renal patients with mild-to-moderate renal insufficiency associates with increased fracture rate [111] thus suggesting its use in these patients. The last point to consider in the intricate pathogenesis of ROD is that in early renal insufficiency the earliest lesion of bone, developing since CKD stages 2 and 3, seems to be a low bone turnover (ABD), secondary to resistance to PTH action or to insufficient PTH response to the ensuing mineral derangements [112]. Thus, high turnover bone should occur only later, in advanced stages of renal failure with long lasting SHPT. As a whole, distinguishing OP from ROD in the early stages of renal insufficiency may be challenging and would possibly require bone biopsy, since therapies may be different for different lesions.

In this intriguing context, the role of native vitamin D is still not addressed but might be significant, since besides reducing the development of SHPT, it could also exert direct effects on bone cells and/or on bone muscles.

## Questions and answers

### 1. What is, if any, the therapeutic role of native vitamin D in renal bone diseases?

Theoretically, vitamin D insufficiency could cause defective mineralization and osteomalacia and invariably carries some degree of SHPT. Therefore, supplementing native vitamin D seems reasonable and, for bone purposes, should predictably carry lower SHPT and less defective mineralization. The absence of evidence in this field is clearly illustrated by a very recent debate specifically aimed at considering the role of native vitamin D supplementation in renal patients [113–115]. The Author for pros [113] underlined that vitamin D is cheap and suggests a somehow holistic prescription that, at reasonably low doses, can be expected to only have the recognized physiological bone effects. In contrast, opposing Author [114] underline that metanalysis of clinical data clearly shows the inconsistency of results of vitamin D supplementation on most of the tested outcomes (including bone), except for the rising of 25-OH-D levels. The Moderator’s opinion [115] is that only a more precise profiling of individuals (by future omics sciences) will possibly provide a definitive answer on the therapeutic effects of native vitamin D.

### 2. Given the recently reported increment of ABD, should we drastically limit vitamin D use in renal patients?

The low number of bone biopsies available in renal patients evidences a rising prevalence of adynamic bone in dialysis patients [116, 117] and an unsuspected high prevalence in conservative renal failure [118, 119]. The reason for this finding is mostly referred to aggressive use of PTH-suppressing therapies (in particular active vitamin D in dialysis [120]) and the occurrence of bone resistance to PTH in non-dialysis [112]. Establishing if native vitamin D may specifically increase ABD is however speculative. Native vitamin D may have suppressive effects on PTH, but in renal patients with limited renal hydroxylation capacity, this could be clinically trivial. A wise opinion could be that moderate replenishment with a natural and cheap compound could predictably avoid significant vitamin D insufficiency and reduce morbidity, possibly through other non-bone direct effects, such as reduction of falls [121].

### 3. Is there any room for double vitamin D metabolites use in renal patients?

To our knowledge, there is no available clinical evidence for a combined (native plus active) vitamin D metabolites use in renal patients. For bone purposes, in particular, we can only speculate that native vitamin D may have a “nutritional” role and avoid deficiency, hence, it will predictably limit the eventual SHPT and limit the risk of defective mineralization. However, we must be aware that native metabolites are ineffective in curing more severe cases of PTH hypersecretion. The activated metabolites (either natural or synthetic) have demonstrated superiority in suppressing PTH and in improving bone lesions, up to the induction of ABD. There is room, then, for randomized clinical trials aiming at finding the most appropriate dose of native metabolite. In the past, very few comparisons have been made, and only one paper is available showing mild advantage with the combined use of 25-OH-D and of 1,25(OH)2D3 in a very low number of dialysis patients evaluated with bone histology [122].

## Vitamin D and rheumatic diseases affecting the kidney

Vitamin D has potential effects on extra-skeletal rheumatic diseases. The latter effects may be explained by: a) the presence of VDRs on different cells other than bone cells such as cartilage cells, synoviocytes and muscle cells; b) vitamin D controls the transcription of genes involved in rheumatic diseases; c) vitamin D has suppressive effects on adaptive immune system and on inflammation via regulating the production of cytokines and the proliferation of cells, both of which are crucial for the pathogenesis of inflammatory diseases, including some rheumatic diseases affecting the kidney [123, 124]. Moreover the independent extrarenal production of calcitriol should be considered to understand the multiple autocrine and paracrine effects of cholecalciferol in patients with CKD [125].

## Questions and answers

### 1. Do you recognize vitamin D to play a role in the modulation of the immune/inflammation system?

Vitamin D as an immune system regulator was suggested in early studies with the discovery of the presence of specific receptors (VDR) in activated T cells [126]. More recent studies have shown that 1,25(OH)2D3 regulates both adaptive and innate immunity but in opposite directions [127–129]. 1,25(OH)2D3 inhibits the adaptive immune response (inducing tolerogenic effects and decreasing autoimmunity risk) and promotes the innate immunity (with relative antimicrobial activity). A statistically significant inverse association between vitamin D status and development of any autoimmune disease was found [130], suggesting a possible protective role of a higher vitamin D status in autoimmune disease. The immunosuppressive effect of 1,25(OH)2D3 is correlated with a decrease in inflammatory cytokines. In fact, for example, in rheumatoid arthritis patients disease activity is inversely correlated to the levels of 25-OH-D [131, 132].

### 2. Vitamin D: a role in systemic lupus erythematosus (SLE) and in particular in its renal involvement?

Since sun exposure in patients with SLE is a possible risk factor for reactivation of the disease, it is not surprising that hypovitaminosis D is very common in these patients [133–140]. It has been observed that 25-OH-D levels are lower in Afro-American women (known to have a high incidence of SLE) than in those living in Sierra Leone, where SLE incidence is low, thus supporting an etiopathogenetic role of hypovitaminosis D in SLE [141]. Associations between VDR polymorphism and SLE have been demonstrated [142], again supporting a role of vitamin D in the pathogenesis and clinical expression of the disease. For example in the Asiatic population a correlation between B allele of Bsml polymorphism and the risk of developing SLE has been demonstrated (OR 3.58, 95%IC: 1.41–9.13, *p* = 0.007) [143]. However, this result was not confirmed in Caucasian populations [142, 143]. It has also been observed that low levels of vitamin D are associated with an increased risk of ANA positivity, lymphocyte B activation, and IFN-α activity [144]. Levels of anti-nDNA antibodies, anti-Smith antibodies and IgG increase as 25-OH-D decreases [145]. As in rheumatoid arthritis [131], there is also evidence of a negative correlation between vitamin D levels and disease activity in adult [146–152] and pediatric SLE patients [153–155]. In particular, a negative correlation between low vitamin D and SLEDAI (SLE disease activity score) has been described [156]. This result may be due to the reversibility of differentiation and maturation of dendritic cells when exposed to calcitriol. Actually, disease reactivation has been demonstrated to be caused by seasonal decrease in vitamin D levels [157]. Vitamin D supplementation in patients with SLE has been studied with the purpose of changing clinical outcomes; however, the results remain inconsistent [158]. Administering a 50,000 IU weekly dose of vitamin D in patients with SLE and hypovitaminosis D [159] has been observed that an increase of 20 ng/mL in 25-OH-D serum levels was associated with a 21% reduction of having a high disease activity index and a 15% reduction in proteinuria.

### Vitamin D in kidney transplanted patients

Reduced 25-OH-D levels are commonly observed in kidney transplant recipients (KTRs) [160]. Persistent SHPT has been reported at 1–2 years and even 5 years after transplantation in 20–50% of cases [161].

Evidence indicates that lowering of PTH levels is safe for bone in KTRs and native vitamin D may be used to lower PTH levels after kidney transplant [162]. However, the effects of ergocalciferol, cholecalciferol and calcifediol on BMD still remains controversial [163–165].

To date, no specific guidelines are available to guide native vitamin D replenishment in KTRs. Furthermore, the dose, administration schedule and 25-OH-D target levels are still a matter of debate in KTRs [166].

## Questions and answers

### 1. Is there evidence that SHPT is associated with increased bone fragility after transplantation or do we consider with caution its correction in these patients?

Alterations in bone metabolism are common after successful renal transplant (RT). Fracture incidence increases markedly in KTRs [167–169], with fracture rates being in the order of 5–44% [170]. Compared to dialysis patients, the short-term (6 months) risk of hip fracture is 34% higher in KTRs [171]. In addition, fractures are associated with increased morbidity and mortality in RT patients compared with the general population [172]. Post-transplantation bone disease is influenced by multiple factors. The negative effect of immunosuppressive therapy on bone [173], diabetic nephropathy, age, gender, dialysis duration, and high or low PTH levels pre-transplantation are considered major causes [170]. However, another important risk factor for bone morbidity seems to be represented by SHPT, that is present in up to 50% of patients even after successful transplantation [169, 174–177]. Several authors have shown that SHPT after RT may negatively affect bone metabolism, thus resulting in bone fragility [169, 174, 175, 178–181]. In a 3-year follow-up study on stable KTRs, Heaf and coworkers [178] found that PTH levels >150 ng/L were predictors of a progressive decrease in BMD, especially at the femoral site. In a long-term study (up to 4 years) on pancreas-kidney transplantation, the decrease in BMD was associated with the presence of SHPT [181]. We showed that PTH values were the most important predictors of high type I collagen cross-linked N-telopeptide (NTx) and low BMD in stable RT subjects [174]. Data on the relationship between SHPT and fracture incidence are rather sparse. However, some authors have reported that high PTH levels may also be responsible for an increased rate in these events. In a study on 125 renal allograft recipients transplanted 44 ± 23 months before, vertebral fractures were detected in 57% of the subjects. PTH and time since transplant were significantly associated with vertebral fractures. However, patients with two or more vertebral fractures showed serum PTH levels >140 pg/mL and these values were 50% higher than patients without fractures, even after multiple adjustment for possible confounders [169]. In a retrospective analysis of 143 RT patients, Perrin et al. found that the PTH threshold for predicting fractures was 130 ng/L. In the same study, a multivariate analysis showed that PTH > 130 ng/L was an independent risk factor for fracture (adjusted hazard ratio [AHR] = 7.5, 95% CI 2.18–25.50) [179]. Some insights into possible beneficial effects on bone health of PTH level-lowering strategies, are possible from some recently published papers. Perrin and coworkers [182] compared 2 groups of patients who consecutively underwent RT 5 years from each other (Group 1, between 2004 and 2006 and Group 2, between 2009 and 2011). The authors reported that, due to some recent improvements in the clinical management of RT patients, SHPT (PTH > 130 ng/L) and bone turnover markers were significantly reduced in Group 2. Accordingly, fracture incidence at 1 year decreased significantly (3.1 vs. 9.1%, *p* = 0.047) in Group 2 compared to Group 1, despite the absence of any steroid sparing in Group 2. Trillini et al. in a randomized prospective study on RT patients, found that paricalcitol treatment was associated with a substantial decrease in PTH levels and, in turn, with a decrease in markers of bone remodeling and BMD loss [183]. A randomized study comparing parathyroidectomy with cinacalcet in RT patients with SHPT and hypercalcemia demonstrated that the decrease in PTH levels was associated with an improvement in femoral BMD [184]. In another small study on the 36-month cinacalcet use in RT patients, a decrease in PTH levels and an increase in BMD vs. control patients was found [185]. Regardless of these considerations, we are still missing robust prospective data on the relationship between SHPT and bone health in RT patients, as well as studies on the effects of PTH lowering strategies on bone status as a primary end-point. However, the large body of evidence currently available strongly suggest that PTH levels >150 pg/L are unsafe for bone and that strategies aimed to control SHPT may decrease bone fragility in RT recipients.

### 2. Do we have sufficient data to support the hypothesis that native vitamin D treatment and the correction of hypovitaminosis D are able to lower PTH levels without using active forms of vitamin D or vitamin D analogs after RT?

As already mentioned, SHPT is a very common feature in RT patients [169, 174–177], with many factors contributing to this phenomenon. Among them, the long-term persistence of parathyroid gland enlargement, the lengthy time required for the involution of parathyroid gland hyperplasia and an alteration in calcium set-point are probably the more relevant [170]. Interestingly, low levels of 25-OH-D have repeatedly been reported in RT patients [160, 169, 186–188]. Indeed, RT subjects may be more susceptible to reduced levels of 25-OH-D because of decreased sun exposure, following recommendation to prevent skin cancers [189–191] and because of increased 25-OH-D catabolism, possibly induced by immunosuppressive drugs and FGF23 [166]. Unfortunately, no randomized controlled trials have been carried out to assess the possible benefits of native vitamin D treatment in this setting, even if some studies are ongoing [164, 192]. It is worth noting that similar to the general population, low 25-OH-D levels are also implicated in the PTH increase in RT patients [165, 169, 188, 193]. Even in this case, the PTH-lowering therapeutical strategy by using cholecalciferol has not been evaluated in robust randomized, placebo-controlled studies. However, the possibility to interfere with PTH hypersecretion by means of native vitamin D, which is cheaper and safer than active vitamin D compounds, has gained attention from several authors. In a study on vitamin D- insufficient RT patients, cholecalciferol administration at a dose of 25,000 IU/monthly plus 400 mg of daily calcium was associated with a large reduction in PTH levels over a 12-month follow-up period [163]. Interestingly, none of the subjects achieving the value of 30 ng/mL of serum 25-OH-D had PTH levels higher than 100 pg/mL. In a small population sample of 14 newly transplanted patients with vitamin D insufficiency, treated with cholecalciferol 400 IU daily plus calcium, 25-OH-D increased significantly, with PTH levels being decreased by half at the end of the 12-month study period [194]. Markedly higher cholecalciferol doses, given in a different administration schedule, were used by Courbebaisse et al. in newly transplanted subjects with low 25-OH-D levels [195]. In a first 3-month intensive phase, 100,000 IU of cholecalciferol were administered every two weeks, followed by a 6-month maintenance period in which the same dose was given every 2 months. PTH levels were stably decreased by cholecalciferol treatment. In none of these studies, cases of hypercalcemia were detected after treatment and urine calcium remained in the normal range. More recently, a short-term study has been carried out using calcifediol at a dose of 266 µg biweekly or monthly in RT patients with very low 25-OH-D serum levels [196]. Both administration schedules were associated with a normalization in vitamin D levels and a significant decrease in PTH values. Even in this case, serum calcium and phosphate remained in the normal range. It is well established that there is a large variability in the characteristics of these studies, including patient selection, length of the follow-up, vitamin D dose and administration schedule. In addition, the data currently available do not provide direct evidence of a benefit in terms of bone health. However, it is reassuring that, irrespective of the differences across the results published so far, native vitamin D administration is associated with a significant decrease in PTH levels, in the absence of important side effects.

### 3. Can the normal range for the general population be used to assess vitamin D status after RT or, rather, do we need specific normal parameters for these patients?

Normal values for serum 25-OH-D are still a matter of debate for the general population [24–26] (see previous section on Vitamin D and PTH levels in the general population). Thus, it is not surprising that truly adequate values of 25-OH-D levels have not been established in RT recipients [166]. The National Kidney Foundation/Kidney Disease Outcomes Quality Initiative (NKF-KDOQI) guidelines recommend vitamin D supplementation when serum 25-OH-D concentration is lower than 30 ng/mL in patients with CKD stage 3 or 4. Accordingly, after transplantation, patients should be treated as non-transplanted CKD patients and with the same levels of GFR [19]. The KDIGO Clinical Practice Guideline for the Diagnosis, Evaluation, Prevention, and Treatment of chronic kidney disease–mineral and bone disorder (CKD–MBD), suggests to treat RT recipients with native vitamin D as it is currently performed for the general population [77]. However, the recommendation was low-graded (2C), based on the substantial lack of specific data in RT patients. Indeed, the main outcomes studied so far to establish the adequacy of calcidiol serum levels in the general population are changes in PTH values, intestinal calcium absorption and bone health and it is well-known that all of these are altered not solely depending on vitamin D status in RT patients [166]. Data specifically aimed to evaluate what can be considered adequate 25-OH-D levels for the prevention of fragility fracture and optimal intestinal calcium absorption in RT patients are completely missing. As reported earlier, some studies have been carried out to evaluate changes in PTH after native vitamin D treatment in RT recipients. Treating RT patients with cholecalciferol, Courbebaisse et al. observed that the large majority of the subjects reached serum PTH concentrations within KDOQI recommended values according to their GFR at 25-OH-D concentration of 30 ng/mL. None of the patients experienced new cases of hypercalcemia or hypercalciuria. In another study in which RT recipients were treated with monthly 25,000 IU of cholecalciferol plus oral calcium, no patient showed iPTH levels higher than 100 pg/mL when 25-OH-D levels were above 30 ng/mL [164], without significant changes in serum and urine calcium. When looking at the possible extra-skeletal effects of native vitamin D in renal transplantation, low vitamin D levels were strongly associated with a decline in eGFR in a cross-sectional study on long-term survivors to RT [197]. Interestingly, patients with sufficient vitamin D levels, defined as 25-OH-D serum values ≥20 ng/mL were significantly protected compared to subjects with vitamin D insufficiency or deficiency. In addition, no eGFR deterioration was seen in subjects with vitamin D levels ≥30 ng/mL. These results were confirmed even after strict adjustment for possible confounders. It remains clear that reliable data for defining whether there is a need for specific normal serum 25-OH-D values are still missing for RT patients. However, based on the results published to date and in the absence of further definitive evidence, clinicians could reasonably interpret 25-OH-D levels in RT subjects as is currently undertaken for the general population.

## Conclusion

Although our knowledge and understanding has improved in terms of negative outcomes and effects associated with low vitamin D status and how treatment with different forms of vitamin D can correct these associated problems, several ongoing issues still need to be resolved in order to more effectively treat patients. A consensus on acceptable cut-off values and on what constitutes insufficient and adequate vitamin D levels is also needed to favouring tailored treatment of patients based on their specific background conditions or disease states. While awaiting for more “solid” data, it is possible to assume that oral vitamin D, such as cholecalciferol, can yield benefits in several clinical settings related to CKD.
